# Ecological differentiation, lack of hybrids involving diploids, and asymmetric gene flow between polyploids in narrow contact zones of *Senecio carniolicus* (syn. *Jacobaea carniolica*, Asteraceae)

**DOI:** 10.1002/ece3.1430

**Published:** 2015-02-22

**Authors:** Karl Hülber, Michaela Sonnleitner, Jan Suda, Jana Krejčíková, Peter Schönswetter, Gerald M Schneeweiss, Manuela Winkler

**Affiliations:** 1 Division of Conservation Biology, Vegetation Ecology and Landscape Ecology, Department of Botany and Biodiversity Research, University of ViennaVienna, Austria; 2 Vienna Institute for Nature Conservation & AnalysesVienna, Austria; 3 Department of Botany, Faculty of Science, Charles University in PraguePrague, Czech Republic; 4 Institute of Botany, The Czech Academy of SciencesPrůhonice, Czech Republic; 5 Institute of Botany, University of InnsbruckInnsbruck, Austria; 6 Division of Systematics and Evolutionary Botany, Department of Botany and Biodiversity Research, University of ViennaVienna, Austria; 7 GLORIA co-ordination, Center for Global Change and Sustainability, University of Natural Resources and Life Sciences ViennaVienna, Austria; Institute for Interdisciplinary Mountain Research, Austrian Academy of SciencesInnsbruck, Austria

**Keywords:** Asymmetric gene flow, contact zone, ecological niche, hybrid cytotypes, polyploidy, *Senecio carniolicus* (Asteraceae)

## Abstract

Areas of immediate contact of different cytotypes offer a unique opportunity to study evolutionary dynamics within heteroploid species and to assess isolation mechanisms governing coexistence of cytotypes of different ploidy. The degree of reproductive isolation of cytotypes, that is, the frequency of heteroploid crosses and subsequent formation of viable and (partly) fertile hybrids, plays a crucial role for the long-term integrity of lineages in contact zones. Here, we assessed fine-scale distribution, spatial clustering, and ecological niches as well as patterns of gene flow in parental and hybrid cytotypes in zones of immediate contact of di-, tetra-, and hexaploid *Senecio carniolicus* (Asteraceae) in the Eastern Alps. Cytotypes were spatially separated also at the investigated microscale; the strongest spatial separation was observed for the fully interfertile tetra- and hexaploids. The three main cytotypes showed highly significant niche differences, which were, however, weaker than across their entire distribution ranges in the Eastern Alps. Individuals with intermediate ploidy levels were found neither in the diploid/tetraploid nor in the diploid/hexaploid contact zones indicating strong reproductive barriers. In contrast, pentaploid individuals were frequent in the tetraploid/hexaploid contact zone, albeit limited to a narrow strip in the immediate contact zone of their parental cytotypes. AFLP fingerprinting data revealed introgressive gene flow mediated by pentaploid hybrids from tetra- to hexaploid individuals, but not vice versa. The ecological niche of pentaploids differed significantly from that of tetraploids but not from hexaploids.

## Introduction

Ecological differentiation is among the most important mechanisms of reproductive isolation among cytotypes of different ploidy (for simplicity termed “cytotypes” from here on) in heteroploid plant species (Levin 1983). It may arise as a direct consequence of genome duplication (Otto and Whitton 2000) or result from subsequent disruptive selection (Petit et al. 1999; Ramsey and Schemske 2002). The degree of ecological differentiation ranges from shifts in the relative abundance of accompanying species (Johnson et al. 2003) via different preferences along ecological gradients within the same habitat type (Raabová et al. 2008) to separation of cytotypes into formations of different physiognomy (Lumaret et al. 1987). Allopolyploids are expected to show stronger ecological differentiation than autopolyploids due to the merging of two differentiated genomes (Soltis and Soltis 2009; Parisod et al. 2010), but there is also evidence for adaptive niche divergence in autopolyploids (Parisod et al. 2010, and references therein).

Contact zones of cytotypes – we use the term in a strict sense to encompass areas of close spatial proximity of individuals of different ploidy – can be observed in many taxa. They provide a unique opportunity to assess isolation mechanisms governing coexistence of cytotypes, such as ecological differentiation (Petit et al. 1999). Major aspects include avoidance of competition and patterns of gene flow between parental cytotypes, potentially leading to long-term coexistence of cytotypes or the formation of new hybrids (Kolář et al. 2009; Hülber et al. 2011). To date, niche differentiation among cytotypes has been assessed by comparing single-cytotype populations (e.g., Manzaneda et al. 2012; McIntyre 2012; Martin and Husband 2013) or by large-scale surveys of the distribution and ecological differentiation of parapatric (Hardy et al. 2000) and sympatric cytotypes (Sonnleitner et al. 2010; Sabara et al. 2013). Patterns of niche differentiation in areas of immediate contact allow inferring whether contact zones represent hybrid zones, that is, habitats suited for both cytotypes, or mosaic zones, that is, a microspatial mixture of habitats each suited for a single cytotype. In the first case, niche differences in contact zones are expected to be smaller compared to both adjacent pure populations and the entire distribution ranges of the cytotypes, whereas no such reduction in niche differences is expected in case of mosaic zones.

The degree of reproductive isolation of cytotypes, that is, the frequency of heteroploid crosses and subsequent formation of viable and (partly) fertile hybrids, plays a crucial role for the long-term integrity of lineages in contact zones (Barton and Hewitt 1985) in general, and for the local maintenance of ploidy variation in particular (Husband et al. 2013; Madlung 2013). For instance, gene flow via individuals of intermediate ploidy may lead to introgression and thus an increase of genetic diversity, transfer of adaptations, or the emergence of new adaptations in the receiving lineage (Soltis and Rieseberg 1986; Rieseberg et al. 1996; Petit et al. 1999). As a consequence, introgressed lineages tend to have broader niches than their pure counterparts (Choler et al. 2004). In heteroploid systems, gene flow and introgression have so far mostly been observed in diploid/tetraploid contact zones (Neuffer et al. 1999; Ståhlberg and Hedrén 2009), while studies on genetic interactions between lineages of higher ploidy are largely lacking for wild species. In two species of *Rorippa* (Brassicaceae), bidirectional introgression between diploids and polyploids (tetra- and hexaploids) was found (Bleeker 2003), but the consequences of introgression for niche evolution were not explored.

Hybrid cytotypes emerging in contact zones face competition with the parental cytotypes. Establishment, persistence, and genetic integrity of hybrid cytotypes will be affected by the magnitude of niche divergence from parental cytotypes, conferring spatial separation and, thereby, reducing competitive interactions and the incidence of heteroploid crosses. Although niche differentiation among cytotypes was documented even in narrow contact zones (Mráz et al. 2012) and odd-ploid hybrid cytotypes were found in many model systems (e.g., Sabara et al. 2013), little is known on niches of hybrid cytotypes and their ecological position relative to their parents. Ståhlberg and Hedrén (2009) reported an intermediate position of triploid hybrids in mixed diploid/tetraploid populations of the *Dactylorhiza maculata* group, albeit without statistical evaluation due to the low number of triploids.

A well-suited system to study mechanisms of ploidy coexistence is the high mountain plant *Senecio carniolicus* (Asteraceae). This species comprises three main cytotypes (diploids, tetraploids, hexaploids) co-occurring in every conceivable combination across the distributional range in the Alps (Sonnleitner et al. 2010). Cytotypes are ecologically differentiated on a large scale (Sonnleitner et al. 2010); so far, only the frequently co-occurring diploids and hexaploids were shown to occupy different niches also on a local scale (Schönswetter et al. 2007; Hülber et al. 2009). Cytotypes show low crossability (crosses between diploids and polyploids) or are interfertile (crosses between polyploids; Sonnleitner et al. 2013), although a range-wide survey of natural populations revealed only low frequencies (< 1%) of hybrid cytotypes (Sonnleitner et al. 2010). Here, we analyze the microspatial, ecological, and genetic structure of narrow contact zones. Specifically, we address the following questions: (1) Does the occurrence of hybrid cytotypes in contact areas correspond to patterns of crossability of cytotypes? (2) Can the ecological differentiation of main cytotypes observed at large spatial scales also be found in areas of immediate contact? Do ecological requirements of hybrids differ from those of the parental cytotypes? (3) What are the patterns of gene flow between the interfertile polyploid cytotypes? Is there evidence for the presence of F2 or later-generation individuals, suggesting at least partial fertility of F1 hybrids? (4) Is there indication for broadening of the ecological niche in introgressed individuals?

## Materials and Methods

### Study species

**Figure 1:**
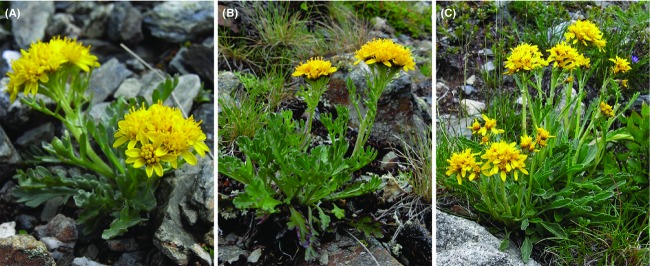
The study species *Senecio carniolicus*: (A) diploid individual, (B) tetraploid individual and (C) hexaploid individual.

*Senecio carniolicus* Willd. (syn. *Jacobaea carniolica* (Willd.) Schrank) is a herbaceous perennial common on acidic bedrock in the alpine to subnival belt of the Eastern Alps and the Carpathians. It constitutes a polyploid complex comprising mainly diploids (2*n* = 2*x* = 40), tetraploids (2*n* = 4*x* = 80), and hexaploids (2*n* = 6*x* = 120) in the Eastern Alps and only hexaploids in the Carpathians (Suda et al. 2007; Sonnleitner et al. 2010). The chromosome number of 40 does not correspond to the diploid level when taking the entire tribe Senecioneae into account but rather represents the lowest number encountered in the “Incani Clade”, where *S. carniolicus* belongs to (Pelser et al. 2003; Escobar García et al. 2012). In contrast to the majority of heteroploid taxa, *S. carniolicus* does not form a single contact zone containing otherwise geographically well-separated cytotypes (Husband and Schemske 1998; Hardy et al. 2000; Mandáková and Münzbergová 2006; Španiel et al. 2008); instead, various combinations of cytotypes occur throughout major parts of the Eastern Alps (Suda et al. 2007; Sonnleitner et al. 2010). Of 100 investigated sample sites, diploids and hexaploids, tetraploids and hexaploids, and diploids and tetraploids co-occur in 28, five, and three sites, respectively, and all three cytotypes co-occur in eight sample sites. Molecular genetic evidence suggests that the polyploid cytotypes are autopolyploid derivatives of a diploid lineage distributed in the easternmost Alps (M. Winkler, G. Pedro Escobar, R. Flatscher, M. Sonnleitner, J. Suda, K. Hülber, P. Schönswetter, G.M. Schneeweiss, unpublished data). Strong genetic divergence between the ancestral eastern diploid lineage and its polyploid derivatives as well as weaker but consistent differentiation between tetraploids and hexaploids renders ongoing polytopic origin of the polyploids unlikely (M. Winkler et al., unpublished data), which is in line with consistent morphological differentiation (Flatscher 2010; Fig.1). Despite substantial habitat segregation (Sonnleitner et al. 2010), individuals of different cytotypes commonly occur in close spatial proximity (less than one meter; Hülber et al. 2009), making *in situ* heteroploid pollination likely.

### Field work

Three mountains with contact zones of two main cytotypes of *S. carniolicus* were selected: Grosser Rosennock (2,265 m a.s.l.; N 46°52′32″, E 13°43′07″): diploids and tetraploids; Sadnig (2,745 m a.s.l.; N 46°56′30″, E 12°59′20″): diploids and hexaploids; and Hoazhöhe (2,275 m a.s.l.; N 46°54′43″, E 13°55′41″): tetraploids and hexaploids. Within a clearly defined cluster comprising approximately 200 plants and surrounded by a non-inhabited area, the spatial position of each studied *S. carniolicus* individual was determined with a laser distance meter (Leica DISTO D5, Leica Geosystems, Heerbrugg, Switzerland). The DNA-ploidy level of all individuals was determined from silica-dried leaf material using flow cytometry (see Sonnleitner et al. 2010 for details); high-resolution histograms (with coefficients of variation of G0/G1 peaks of *S. carniolicus* samples below 3%) were achieved in more than 92% of analyses. Presence of vascular plant species occurring within a radius of 0.2 m around each *Senecio* individual was recorded; data for Sadnig were taken from Hülber et al. (2009).

### DNA extraction, AFLP fingerprinting, and data analysis

Of all individuals sampled in the tetraploid/hexaploid contact zone, total genomic DNA was extracted from similar amounts of dried tissue (ca. 10 mg) with the DNeasy 96 plant mini kit (Qiagen, Hilden, Germany) following the manufacturer's protocol. The AFLP procedure followed Escobar García et al. (2012). Six plants were extracted twice to test the reproducibility of AFLP fingerprinting (Bonin et al. 2004). From the restriction/ligation step onwards, 13 samples were replicated twice, and seven samples were used as replicates between PCR plates, and therefore replicated in every plate. Fragments were scored manually using Genographer 1.6 (version no longer available).

The error rate was calculated as the ratio of mismatches (scoring of 0 vs. 1) over phenotypic comparisons in AFLP profiles of replicated individuals (Bonin et al. 2004). Nonreproducible fragments were excluded from the analyses. Monomorphic fragments and those present/absent in all but one individual were removed from the dataset to avoid biased parameter estimates (Bonin et al. 2004). Intercytotype gene flow was inferred with NewHybrids (version 1.1beta; Anderson and Thompson 2002; Anderson 2008), which allows for the accommodation of dominant multilocus markers such as AFLPs (Anderson 2008). The posterior probability that each sampled individual belongs to each of several classes (parents, F1 and F2 hybrids, backcrosses) is computed by Markov chain Monte Carlo (MCMC) in a Bayesian model-based clustering framework. The probability of class membership was computed with the default settings, without prior information on hybrid status, and using 1.3 million generations following a burn-in of 100,000 generations.

### Analyses of ecological data

Characterization of environmental conditions around sampled individuals was achieved via unweighted mean Landolt indicator values (Landolt 2010) of all vascular plant species (except *Senecio carniolicus*) per circular plot of 0.2 m radius. Landolt indicator values describe ecological requirements of species in terms of temperature (T), light (L), soil moisture (F), soil reaction (R), nutrients (N) and soil humus content (H), and range from 1 (low) to 5 (high). Niche differences among cytotypes in contact zones were tested by comparing mean indicator values among cytotypes using a multivariate analysis of variance (MANOVA). A principal component analysis (PCA) using the same indicator values but standardized to zero mean and unit variance was applied to attain a graphical illustration of the cytotypes’ niches. Spatial aggregation/segregation of cytotypes was tested via Mantel tests correlating a pairwise cytotype “distance” among individuals (0 and 1 for the same and for different ploidy, respectively) with the geographic distances; Kendall's tau coefficient was statistically evaluated by 999 randomizations. All analyses were carried out in R (R Development Core Team 2011). PCA and Mantel test were calculated using the functions dudi.pca (package ade4: Dray and Dufour 2007) and mantel (package vegan: Oksanen et al. 2013), respectively. The package plotrix (Lemon 2006) was used for graphical representations.

A Monte Carlo randomization technique was applied to test whether the niche differences in the contact zones are smaller than those observed across the Eastern Alps (Sonnleitner et al. 2010). The empirical *F*-value of the MANOVA test for niche differentiation in the contact zone was compared against a null distribution of *F*-values generated from randomly chosen individuals of the corresponding cytotypes from the aforementioned survey; the sample size in each of the 9999 permutations equals the number of individuals per cytotype in the contact zone. All analyses were performed separately for each of the three contact zones.

## Results

**Figure 2:**
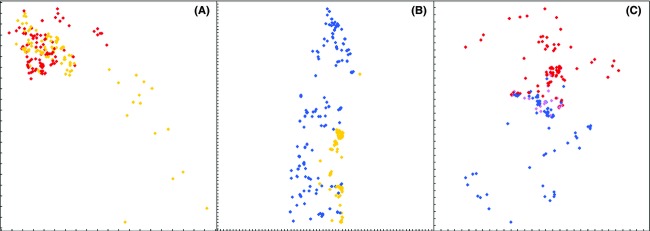
Spatial distribution of cytotypes of *Senecio carniolicus* in three narrow contact zones: (A) diploid/tetraploid (Rosennock), (B) diploid/hexaploid (Sadnig), and (C) tetraploid/hexaploid (Hoazhöhe). Yellow, red, blue, and violet dots represent diploid, tetraploid, hexaploid, and pentaploid individuals. Ticks are at 1 m distances.

A total of 181, 275 and 190 individuals were recorded in the three contact zones Rosennock (diploid/tetraploid), Sadnig (diploid/hexaploid) and Hoazhöhe (tetraploid/hexaploid), respectively. In the diploid/tetraploid and in the diploid/hexaploid contact zones, no individuals with the expected intermediate ploidy were found; one pentaploid plant found in the diploid/hexaploid contact zone most likely arose because of the involvement of an unreduced gamete of the diploid and was disregarded in further analyses. In contrast, within the tetraploid/hexaploid contact zone, 26 pentaploid individuals were observed. Mantel tests revealed highly significant spatial clustering of main cytotypes in the contact zones (Table1; *P *= 0.001 for each pairwise comparison). Pentaploids were spatially significantly separated from tetraploids (*P *= 0.044), but not from hexaploids (*P *= 0.816). diploids and hexaploids showed stronger spatial clustering in their contact zone than diploids and tetraploids (Fig.2; Table1). Tetra- and hexaploids formed largely pure clusters, but pentaploids were intermixed with both parental cytotypes (Fig.2). Pentaploids were restricted to a narrow, 6- to 7-m-wide strip at the immediate contact of tetra- and hexaploids, where the three cytotypes were approximately equally abundant (Fig.2).

**Table 1:** Ecological differentiation and spatial clustering of cytotypes of *Senecio carniolicus* in three narrow contact zones. Pillai refers to the Pillai–Bartlett trace test statistic. Subscripts for the *F*-value give the numerator and denominator degree of freedom

	Ecological differentiation (MANOVA)			Spatial clustering (Mantel test)	
	Pillai	*F*-value	*P* value	*r*	*P* value
Rosennock					
Diploids _(90)_ / tetraploid _(91)_	0.12	*F*_6, 174 _= 3.98	**<0.001**	0.033	**0.001**
Sadnig					
Diploids _(110)_ / hexaploid _(165)_	0.43	*F*_6, 268 _= 33.91	**<0.001**	0.127	**0.001**
Hoazhöhe					
Tetra- _(90)_ / hexaploid _(74)_	0.31	*F*_6, 157 _= 11.57	**<0.001**	0.273	**0.001**
Tetra- _(90)_ / pentaploid _(26)_	0.22	*F*_6, 109 _= 5.18	**<0.001**	0.076	**0.044**
Penta- _(26)_ / hexaploid _(74)_	0.10	*F*_6, 93 _= 1.78	0.111	−0.038	0.816

Results were obtained by multivariate analyses of variance (MANOVA) using mean Landolt indicator values (Landolt 2010) of accompanying vascular plant species and by Mantel tests performing 999 permutations. Values in parentheses indicate the number of individuals. Significant differences between ploidies are given in bold.

**Figure 3:**
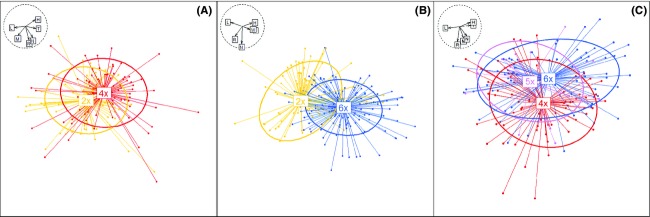
Ecological niches of cytotypes of *Senecio carniolicus* in three narrow contact zones: (A) diploid/tetraploid (Rosennock), (B) diploid/hexaploid (Sadnig), and (C) tetraploid/hexaploid (Hoazhöhe). Values were derived from principal component analyses (PCAs) using Landolt indicator values (Landolt 2010) of vascular plant species accompanying target individuals. Confidence ellipses are defined by the centroid and the standard deviation of the cloud. Ordination axes represent 43% (*x*-axis) and 26% (*y*-axis;), 50% and 22% as well as 49% and 27% of the explained variance in A, B, and C, respectively. Arrows in the dashed circle (*r* = 1) represent direction and magnitude of effects of environmental variables (eigenvectors of the covariance matrix) represented by the Landolt indicator values for temperature (T), light (L), soil moisture (F), soil reaction (R), nutrients (N), and soil humus content (H). The labels 2*x*, 4*x*, 5*x,* and 6*x* represent centers of niches of diploid (yellow), tetraploid (red), pentaploid (violet), and hexaploid (blue) individuals, respectively.

**Figure 4:**
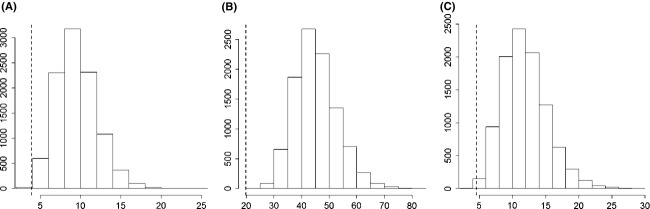
Niche differentiation among cytotypes of *Senecio carniolicus* compared between three narrow contact zones (A: diploid/tetraploid; B: diploid/hexaploid; C: tetraploid/hexaploid) and the overall distribution range of cytotypes in the Eastern Alps. The *x*-axis represents *F*-values derived from multivariate analyses of variance (MANOVA) testing for pairwise niche differentiation between cytotypes, while the *y*-axis represents their frequency. The dashed line and the histogram are the empirical value in the contact zone and the distribution of simulated values derived from a Monte Carlo randomization applying 9999 permutations. Differences were highly significant (*P *= 0.004, *P *< 0.001 and *P *= 0.003, respectively).

The main cytotypes were ecologically highly significantly differentiated (Table1). The strongest contrast was found in the diploid/hexaploid contact zone followed by the tetraploid/hexaploid zone, while the weakest contrast was between diploids and tetraploids (Fig.3). The niche of pentaploids was significantly different from tetraploids, but not from hexaploids. Niche differentiation between main cytotypes was significantly lower in the three contact zones than in their overall distribution (*P *= 0.004, *P* < 0.001 and *P* = 0.003 for the comparison of diploids/tetraploids, diploids/hexaploids, and tetraploids/hexaploids, respectively; Fig.4).

**Figure 5:**
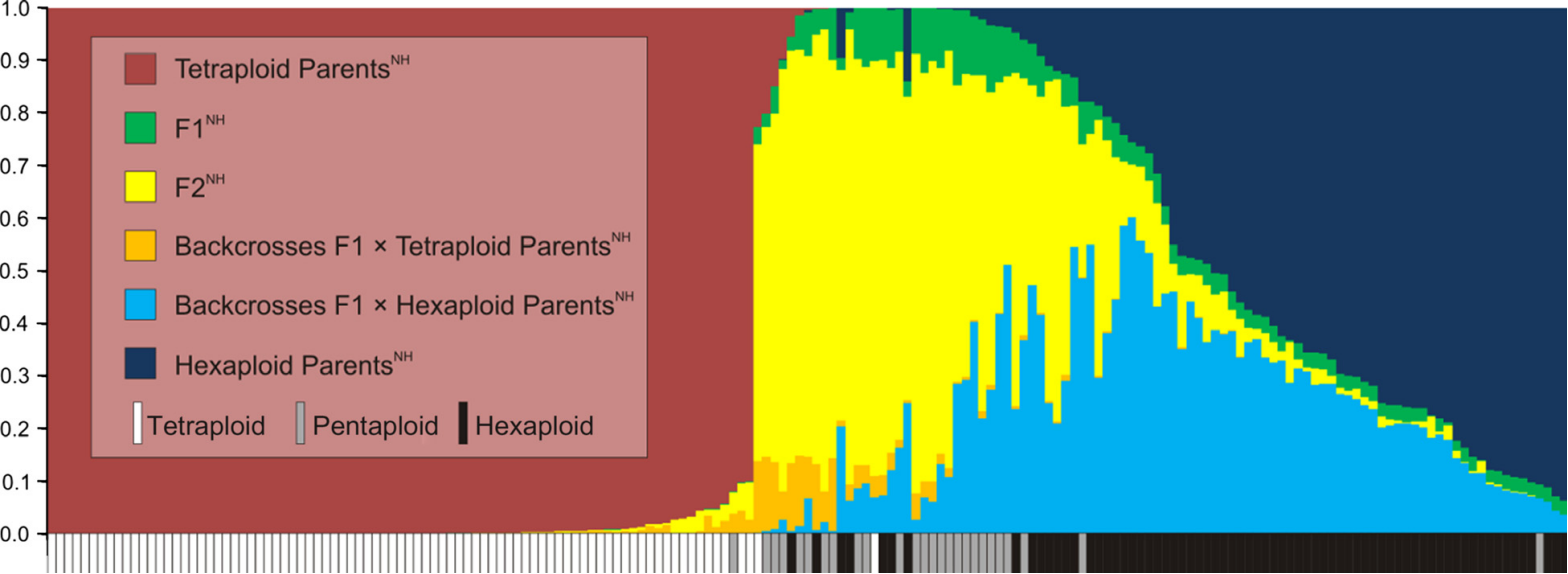
Posterior probability distribution of hybrid classes in a contact zone of tetraploid and hexaploid *Senecio carniolicus* obtained from analysis of AFLP data with the software NewHybrids (Anderson and Thompson 2002; Anderson 2008).

The three AFLP primer combinations yielded 131 unambiguous polymorphic fragments after the removal of 14 nonreproducible, four singular, and 101 homogeneous markers. Seven individuals with nonreproducible AFLP profiles were removed from the dataset, resulting in a total of 183 analyzed individuals. In the AFLP profiles from replicated samples, 451 differences were observed of 14,250 phenotypic comparisons, resulting in an error rate of 3.16%. Almost all tetraploid individuals were classified as Tetraploid Parents^NH^ (hybrid classes suggested by NewHybrids are marked by capitalization and the superscript ^“NH”^), and pentaploids were predominantly classified as F2^NH^. In contrast, less than two-thirds of the hexaploid individuals were Hexaploid Parents^NH^, the remaining ones falling into classes F2^NH^ and Backcrosses F1 ×  Hexaploid Parents^NH^. None of the individuals was categorized as F1^NH^ (Table2). The mean posterior probability of class membership of an individual was highest for Tetraploid Parents^NH^. Penta- and hexaploid individuals showed a highly admixed class membership (Fig.5).

**Table 2:** Membership of *Senecio carniolicus* individuals in a tetraploid/hexaploid contact zone to hybrid classes as identified by the software NewHybrids (Anderson and Thompson 2002; Anderson 2008) based on AFLP fingerprints

Hybrid class	Cytotype		
	Tetraploid	Pentaploid	Hexaploid
Tetraploid Parents^NH^	**97.7 (84)**	3.8 (1)	0 (0)
Hexaploid Parents^NH^	0 (0)	3.8 (1)	**64.8 (46)**
F1^NH^	0 (0)	0 (0)	0 (0)
F2^NH^	2.3 (2)	**84.7 (22)**	16.9 (12)
Backcrosses F1 × Tetraploid Parents^NH^	0 (0)	0 (0)	0 (0)
Backcrosses F1 × Hexaploid Parents^NH^	0 (0)	7.7 (2)	18.3 (13)

Values represent the percentage of individuals (number of individuals in parentheses) for each cytotype with predominant posterior probability of membership to a specific hybrid class. The most frequent hybrid class in each cytotype is given in bold.

The niches of introgressed hexaploids (i.e., those identified as F2^NH^ and Backcrosses F1 ×  Hexaploid Parents^NH^) were slightly wider (mean distance ± SE of each item to PCA class centroid: 2.55 ± 0.14) than those of Hexaploid Parents^NH^ (2.23 ± 0.10), but these differences were not significant (MANOVA: F_6, 64_ = 0.12, *P* = 0.217). Niches of both introgressed hexaploids (F_6, 108_ = 8.54, *P* < 0.001) and Hexaploid Parents^NH^ (F_6, 129_ = 7.09, *P* < 0.001) were highly significantly differentiated from the niche of Tetraploid Parents^NH^.

## Discussion

The spatial distribution of cytotypes provides valuable insights into evolutionary processes shaping polyploid complexes. In a mixed-ploidy population – independent of its origin as primary or secondary hybrid zone – the frequency-dependent mating disadvantage (Felber-Girard et al. 1996) will progressively remove the less frequent cytotype unless a sufficient degree of assortative mating ensures its integrity (Levin 1975; Fowler and Levin 1984; Rodríguez 1996). Among others, lineage integrity may be fostered by microspatial segregation based on divergent ecological preferences. In accordance with these expectations, the three main cytotypes present in Eastern Alpine populations of the heteroploid mountain plant *Senecio carniolicus,* which exhibit individual biogeographic patterns across their distribution range (Sonnleitner et al. 2010) and spatial segregation at the population level (Schönswetter et al. 2007), are spatially separated also at the microscale (Fig.2; Table1). This is in line with other recent studies showing nonrandom spatial patterns in *Ranunculus adoneus* (Baack 2004), *Knautia arvensis* (Kolář et al. 2009), *Allium oleraceum* (Šafářová and Duchoslav 2010), or *Gymnadenia conopsea* (Trávníček et al. 2011). Interfertile cytotypes are expected to be strongly clustered in contact zones, whereas incompatible cytotypes may be less segregated (e.g., Castro et al. 2012). Supporting these expectations, we found the strongest spatial separation for the fully interfertile tetraploids and hexaploids (Table1). For the incompatible diploids and polyploids, other factors such as the fine-grained mosaic of alpine habitats caused by steep abiotic gradients or spatial autocorrelation as a consequence of leptokurtic dispersal kernels skewed toward short dispersal (Nathan and Muller-Landau 2000) may be of greater relevance. However, we cannot exclude that these factors contribute to the strong segregation of tetra- and hexaploids.

Diploid, tetraploid, and hexaploid *S. carniolicus* showed highly significant niche differences within the pairwise contact zones (Table1; Fig.3). Such ecological differentiation at the microscale was also found for di- and autotetraploid *Anthoxanthum alpinum* (Felber-Girard et al. 1996), *Dactylorhiza maculata* (Ståhlberg and Hedrén 2009), and *Chamerion angustifolium* (Martin and Husband 2013), for di- and allotetraploid *Centaurea stoebe* (Mráz et al. 2012), as well as for diploid, tetraploid, and hexaploid *Solidago altissima* (Richardson and Hanks 2011). In contrast, Halverson et al. (2008) found a random spatial pattern suggesting no habitat preferences, and Hanzl et al. (2014) detected no ecological shifts between diploid and autotetraploid *Knautia*. Along the same line, Keeler (1992) identified no relationship between cytotype and grazing, burning regime, or water availability in *Andropogon gerardii*. Ecological differentiation among cytotypes of *S. carniolicus* was significantly weaker in the contact zones than across their entire distribution ranges in the Eastern Alps (Fig.4). This is likely due to the more restricted amplitude of ecological gradients on this small spatial scale, where historic and biogeographic effects are expected to be marginal or lacking (Wiens and Donoghue 2004). Despite introgressive gene flow from tetraploids to hexaploids mediated by pentaploid hybrids (Fig.5) – direct gene flow is unlikely because the ploidy of unreduced and reduced gametes of the lower and higher ploid cytotype, respectively, do not match as in diploid/tetraploid systems – we found no evidence for niche convergence in the tetraploid/hexaploid contact zone. Niche differentiation between tetraploids and hexaploids was intermediate between the stronger and weaker differentiation of diploids and hexaploids, and of diploids and tetraploids, respectively; this pattern remained stable when only introgressed hexaploids (F2^NH^, Backcrosses F1 ×  Hexaploid Parents^NH^; Fig.5) were considered.

Occurrence of hybrid cytotypes differed strongly among the contact zones and agrees well with patterns of cross compatibility (Sonnleitner et al. 2013). Not a single triploid or tetraploid plant was found in the diploid/tetraploid and diploid/hexaploid contact zones, whereas pentaploid hybrids were frequent in the tetraploid/hexaploid contact zone (Fig.2). Reproductive barriers between diploid and polyploid plants appear to be common (e.g., Castro et al. 2012; Münzbergová et al. 2013), whereas different polyploid cytotypes might be interfertile to some extent (Schneider 1958). Pentaploid individuals, which are vigorous, flower regularly, and set well-developed seeds (M. Winkler and M. Sonnleitner, personal observation), were limited to a narrow, a few meters wide strip in the immediate contact zone of their fully interfertile parental cytotypes (Fig.2C). This spatial restriction together with the scarcity of contact areas between tetraploids and hexaploids most likely explains the low frequency (∽0.7%) of pentaploids in a survey of 100 populations throughout the Eastern Alps (Sonnleitner et al. 2010).

Although meiotic irregularities should strongly restrict the reproduction of pentaploids as aneuploid seeds are frequently inviable or at least less viable than euploid ones (Comai 2005), AFLP fingerprinting data revealed introgressive gene flow mediated by pentaploid hybrids from tetraploid to hexaploid individuals, but not vice versa (Fig.5). Presence of primary (F1^NH^) hybrids was not supported, probably indicating that their establishment – despite the interfertility of the two polyploid cytotypes – did not occur recently and may be connected to specific ecological conditions or disturbances (Levin et al. 1996). In contrast, we detected a high number of second-generation hybrids, that is F2^NH^ (i.e., F1 × F1 crosses) as well as Backcrosses F1 ×  Hexaploid Parents^NH^ (Fig.5). Preliminary data from flow cytometric seed screening (J. Suda, unpublished data) revealed that pentaploids yield embryos with hexaploid and aneuploid (DNA contents being intermediate between tetraploids and pentaploids) genome size lending support to the pattern of gene flow suggested by genetics. Aneuploid offspring of pentaploids was frequently found in a putative hybrid zone of tetraploid *Knautia arvensis* and hexaploid *K. dipsacifolia* (Kolář et al. 2009).

Due to the combination of diverged genomes, ecological amplitudes of homoploid hybrids tend to differ from those of their parents (Rieseberg 1997). The hybrid niche is not necessarily intermediate but might also transcend parental attributes due to transgressive segregation (Rieseberg et al. 1999). In heteroploid species, newly generated polyploids might be preadapted to occupy novel habitats (Levin 2003), but studies exploring the niches of hybrid cytotypes in sexual plants are scarce. Only Ståhlberg and Hedrén (2009) estimated the niche of triploids found at low frequencies in diploid/tetraploid contact zones of *Dactylorhiza maculata* s.l. as intermediate between those of the parental cytotypes. The ecological niche of pentaploid *S. carniolicus* differs significantly from that of tetraploids but not from that of hexaploids, albeit being somewhat narrower, resulting in broad overlap with the hexaploid cytotype's niche (Fig.3C). The nontransgressive state of the pentaploid cytotype's niche might contribute to its restricted spatial distribution in close proximity of the parental cytotypes. Moreover, the ecological resemblance of pentaploids to hexaploids indicates that introgression in *S. carniolicus* has not (yet) resulted in adaptive evolution, contrasting previous observations of transfer of adaptations through hybridization in various plant species (Arnold 2004; Martin et al. 2006; Kim et al. 2008; Whitney et al. 2010).

## Data Accessibility

The AFLP data matrix and the matrix of accompanying species along with mean Landolt indicator values for each Senecio carniolicus individual are available at Dryad: doi: 10.5061/dryad.dh178.

## References

[b1] Anderson EC (2008). Bayesian inference of species hybrids using multilocus dominant genetic markers. Philos. Trans. R. Soc. Lond. B Biol. Sci.

[b2] Anderson EC, Thompson EA (2002). A model-based method for identifying species hybrids using multilocus genetic data. Genetics.

[b3] Arnold ML (2004). Transfer and origin of adaptations through natural hybridization: Were Anderson and Stebbins right?. Plant Cell.

[b4] Baack EJ (2004). Cytotype segregation on regional and microgeographic scales in snow buttercups (*Ranunculus adoneus*: Ranunculaceae). Am. J. Bot.

[b5] Barton NH, Hewitt GM (1985). Analysis of hybrid zones. Annu. Rev. Ecol. Syst.

[b6] Bleeker W (2003). Hybridization and *Rorippa austriaca* (Brassicaceae) invasion in Germany. Mol. Ecol.

[b7] Bonin A, Bellemain E, Bronken Eidesen P, Pompanon F, Brochmann C, Taberlet P (2004). How to track and assess genotyping errors in population genetics studies. Mol. Ecol.

[b8] Castro S, Loureiro J, Procházka T, Münzbergová Z (2012). Cytotype distribution at a diploid-hexaploid contact zone in *Aster amellus* (Asteraceae). Ann. Bot.

[b9] Choler P, Erschbamer B, Tribsch A, Gielly L, Taberlet P (2004). Genetic introgression as a potential to widen a species’ niche: insights from alpine *Carex curvula*. Proc. Natl. Acad. Sci. USA.

[b10] Comai L (2005). The advantages and disadvantages of being polyploid. Nat. Rev. Genet.

[b11] Dray S, Dufour AB (2007). The ade4 package: Implementing the duality diagram for ecologists. J. Stat. Softw.

[b12] Escobar García P, Winkler M, Flatscher R, Sonnleitner M, Rauchová J, Suda J (2012). Long-term range persistence in peripheral and interior refugia characterize Pleistocene range dynamics in a widespread Alpine plant species (*Senecio carniolicus*, Asteraceae). Mol. Ecol.

[b13] Felber-Girard M, Felber F, Buttler A (1996). Habitat differentiation in a narrow hybrid zone between diploid and tetraploid *Anthoxanthum alpinum*. New Phytol.

[b14] Flatscher R (2010).

[b15] Fowler NL, Levin DA (1984). Ecological constraints on the establishment of a novel polyploid in competition with its diploid progenitor. Am. Nat.

[b16] Halverson K, Heard SB, Nason JD, Stireman JO (2008). Origins, distribution, and local co-occurrence of polyploid cytotypes in *Solidago altissima* (Asteraceae). Am. J. Bot.

[b17] Hanzl M, Kolář F, Nováková D, Suda J (2014). Nonadaptive processes governing early stages of polyploid evolution: insights from a primary contact zone of relict serpentine *Knautia arvensis* (Caprifoliaceae). Am. J. Bot.

[b18] Hardy OJ, Vanderhoeven S, De Loose M, Meerts P (2000). Ecological, morphological and allozymic differentiation between diploid and tetraploid knapweeds (*Centaurea jacea*) from a contact zone in the Belgian Ardennes. New Phytol.

[b19] Hülber K, Sonnleitner M, Flatscher R, Berger A, Dobrovsky R, Niesser S (2009). Ecological segregation drives fine-scale cytotype distribution of *Senecio carniolicus* in the Eastern Alps. Preslia.

[b20] Hülber K, Berger A, Gilli C, Hofbauer M, Patek M, Schneeweiss GM (2011). No evidence for a role of competitive capabilities of adults in causing habitat segregation of diploid and hexaploid *Senecio carniolicus* (Asteracaeae). Alp. Bot.

[b21] Husband BC, Schemske DW (1998). Cytotype distribution at a diploid-tetraploid contact zone in *Chamerion**Epilobium**angustifolium* (Onagraceae). Am. J. Bot.

[b22] Husband BC, Baldwin SJ, Leitch IJ, Greilhuber J, Doležel J, Wendel JF, Suda J (2013). The incidence of polyploidy in natural plant populations: major patterns and evolutionary processes. Plant genome diversity Volume 2: Physical structure, behaviour and evolution of plant genomes.

[b23] Johnson MTJ, Husband BC, Burton TL (2003). Habitat differentiation between diploid and tetraploid *Galax urceolata* (Diapensiaceae). Int. J. Plant Sci.

[b24] Keeler KH (1992). Local polyploid variation in the native prairie grass *Andropogon gerardii*. Am. J. Bot.

[b25] Kim M, Cui M-L, Cubas P, Gillies A, Lee K, Chapman MA (2008). Regulatory genes control a key morphological and ecological trait transferred between species. Science.

[b26] Kolář F, Štech M, Trávníček P, Rauchová J, Urfus T, Vít P (2009). Towards resolving the *Knautia arvensis* agg. (Dipsacaceae) puzzle: primary and secondary contact zones and ploidy segregation at landscape and microgeographic scales. Ann. Bot.

[b27] Landolt E (2010). Flora indicativa: Ökologische Zeigerwerte und biologische Kennzeichen zur Flora der Schweiz und der Alpen. Ecological indicator values and biological attributes of the Flora of Switzerland and the Alps.

[b28] Lemon J (2006). Plotrix: a package in the red light district of R. R-News.

[b29] Levin DA (1975). Minority cytotype exclusion in local plant populations. Taxon.

[b30] Levin DA (1983). Polyploidy and novelty in flowering plants. Am. Nat.

[b31] Levin DA (2003). The ecological transition in speciation. New Phytol.

[b32] Levin DA, Francisco-Ortega J, Jansen RK (1996). Hybridization and the extinction of rare plant species. Conserv. Biol.

[b33] Lumaret R, Guillerm JL, Delay J, Ait Lhaj Loutfi A, Izco J, Jay M (1987). Polyploidy and habitat differentiation in *Dactylis glomerata* L. from Galicia (Spain). Oecologia.

[b34] Madlung A (2013). Polyploidy and its effect on evolutionary success: old questions revisited with new tools. Heredity.

[b35] Mandáková T, Münzbergová Z (2006). Distribution and ecology of cytotypes of the *Aster amellus* aggregates in the Czech Republic. Ann. Bot.

[b36] Manzaneda AJ, Rey PJ, Bastida JM, Weiss-Lehman C, Raskin E, Mitchell-Olds T (2012). Environmental aridity is associated with cytotype segregation and polyploidy occurrence in *Brachypodium distachyon* (Poaceae). New Phytol.

[b37] Martin SL, Husband BC (2013). Adaptation of diploid and tetraploid *Chamerion angustifolium* to elevation but not local environment. Evolution.

[b38] Martin NH, Bouck AC, Arnold ML (2006). Detecting adaptive trait introgression between *Iris fulva* and *I. brevicaulis* in highly selective field conditions. Genetics.

[b39] McIntyre PJ (2012). Polyploidy associated with altered and broader ecological niches in the *Claytonia perfoliata* (Portulacaceae) species complex. Am. J. Bot.

[b40] Mráz P, Španiel S, Keller A, Bowmann G, Farkas A, Šingliarová B (2012). Anthropogenic disturbance as a driver of microspatial and microhabitat segregation of cytotypes of *Centaurea stoebe* and cytotype interactions in secondary contact zones. Ann. Bot.

[b41] Münzbergová Z, Šurinová M, Castro S (2013). Absence of gene flow between diploids and hexaploids of *Aster amellus* at multiple spatial scales. Heredity.

[b42] Nathan R, Muller-Landau HC (2000). Spatial patterns of seed dispersal, their determinants and consequences for recruitment. Trends Ecol. Evol.

[b43] Neuffer B, Auge H, Mesch H, Amarell U, Brandl R (1999). Spread of violets in polluted pine forests: morphological and molecular evidence for the ecological importance of interspecific hybridization. Mol. Ecol.

[b44] Oksanen J, Blanchet FG, Kindt R, Legendre P, Minchin PR, O'Hara RB (2013). http://CRAN.R-project.org/package<vegan.

[b45] Otto SP, Whitton J (2000). Polyploid incidence and evolution. Annu. Rev. Genet.

[b46] Parisod C, Holderegger R, Brochmann C (2010). Evolutionary consequences of autopolyploidy. New Phytol.

[b47] Pelser P, Gravendeel B, van der Meijden R (2003). Phylogeny reconstruction in the gap between too little and too much divergence: the closest relatives of *Senecio jacobaea* (Asteraceae) according to DNA sequences and AFLPs. Mol. Phylogenetics Evol.

[b48] Petit C, Bretagnolle F, Felber F (1999). Evolutionary consequences of diploid-polyploid hybrid zones in wild species. Trends Ecol. Evol.

[b49] R Development Core Team (2011). R: A language and environment for statistical computing.

[b50] Raabová J, Fischer M, Münzbergová Z (2008). Niche differentiation between diploid and hexaploid *Aster amellus*. Oecologia.

[b51] Ramsey J, Schemske DW (2002). Neopolyploidy in flowering plants. Annu. Rev. Ecol. Syst.

[b53] Richardson ML, Hanks LM (2011). Differences in spatial distribution, morphology, and communities of herbivorous insects among three cytotypes of *Solidago altissima* (Asteraceae). Am. J. Bot.

[b54] Rieseberg LH (1997). Hybrid origins of plant species. Annu. Rev. Ecol. Syst.

[b55] Rieseberg LH, Sinervo B, Linder CR, Ungerer MC, Arias DM (1996). Role of gene interactions in hybrid speciation: evidence from ancient and experimental hybrids. Science.

[b56] Rieseberg LH, Archer MA, Wayne RK (1999). Transgressive segregation, adaptation and speciation. Heredity.

[b57] Rodríguez DJ (1996). A model for the establishment of polyploidy in plants: viable but infertile hybrids, iteroparity, and demographic stochasticity. J. Theor. Biol.

[b58] Sabara HA, Kron P, Husband BC (2013). Cytotype coexistence leads to triploid hybrid production in a diploid-tetraploid contact zone of *Chamerion angustifolium* (Onagraceae). Am. J. Bot.

[b59] Šafářová L, Duchoslav M (2010). Cytotype distribution in mixed populations of polyploid *Allium oleraceum* measured at a microgeographic scale. Preslia.

[b60] Schneider I (1958). Zytogenetische Untersuchungen an Sippen des Polyploid-Komplexes *Achillea millefolium* L. s. lat. (Zur Phylogenie der Gattung *Achillea*, I). Öster. Bot. Zeitschr.

[b61] Schönswetter P, Lachmayer M, Lettner C, Prehsler D, Rechnitzer S, Reich DS (2007). Sympatric diploid and hexaploid cytotypes of *Senecio carniolicus* (Asteraceae) in the Eastern Alps are separated along an altitudinal gradient. J. Plant. Res.

[b62] Soltis DE, Rieseberg LH (1986). Autopolyploidy in *Tolmiea menziesii* (Saxifragaceae) – genetic insights from enzyme electrophoresis. Am. J. Bot.

[b63] Soltis PS, Soltis DE (2009). The role of hybridization in plant speciation. Annu. Rev. Plant Biol.

[b64] Sonnleitner M, Flatscher R, Escobar García P, Rauchová J, Suda J, Schneeweiss GM (2010). Distribution and habitat segregation on different spatial scales among diploid, tetraploid and hexaploid cytotypes of *Senecio carniolicus* (Asteraceae) in the Eastern Alps. Ann. Bot.

[b65] Sonnleitner M, Weis B, Flatscher R, Escobar García P, Suda J, Krejčíková J (2013). Parental ploidy strongly affects offspring fitness in heteroploid crosses among three cytotypes of autopolyploid *Jacobaea carniolica* (Asteraceae). PLoS One.

[b66] Španiel S, Marhold K, Hodálová I, Lihová J (2008). Diploid and tetraploid cytotypes of *Centaurea stoebe* (Asteraceae) in Central Europe: morphological differentiation and cytotype distribution patterns. Folia Geobot.

[b67] Ståhlberg D, Hedrén M (2009). Habitat differentiation, hybridization and gene flow patterns in mixed populations of diploid and autotetraploid *Dactylorhiza maculata* s.l. (Orchidaceae). Evol. Ecol.

[b68] Suda J, Weiss-Schneeweiss H, Tribsch A, Schneeweiss GM, Trávníčcek P, Schönswetter P (2007). Complex distribution patterns of di-, tetra-, and hexaploid cytotypes in the European high mountain plant *Senecio carniolicus* (Asteraceae). Am. J. Bot.

[b69] Trávníček P, Kubátová B, Čurn V, Rauchová J, Krajníková E, Jersáková J (2011). Remarkable coexistence of multiple cytotypes of the *Gymnadenia conopsea* aggregate (the fragrant orchid): evidence from flow cytometry. Ann. Bot.

[b70] Whitney KD, Randell RA, Rieseberg LH (2010). Adaptive introgression of abiotic tolerance traits in the sunflower *Helianthus annuus*. New Phytol.

[b71] Wiens JJ, Donoghue MJ (2004). Historical biogeography, ecology and species richness. Trends Ecol. Evol.

